# In vitro response of vanilla (*Vanilla planifolia* Jacks. ex Andrews) to PEG-induced osmotic stress

**DOI:** 10.1038/s41598-021-02207-0

**Published:** 2021-11-19

**Authors:** Eduardo Martínez-Santos, Carlos Alberto Cruz-Cruz, José Luis Spinoso-Castillo, Jericó Jabín Bello-Bello

**Affiliations:** 1 Colegio de Postgraduados Campus Córdoba, Carretera Córdoba Veracruz, Amatlán de los Reyes Km 348, 94946 Veracruz, Mexico; 2 Universidad Veracruzana-Facultad de Ciencias Químicas, Oriente 6, No. 1009, Orizaba, 94340 Veracruz, Mexico; 3 CONACYT-Colegio de Postgraduados Campus Córdoba, Carretera Córdoba Veracruz, Amatlán de los Reyes Km 348, 94946 Veracruz, Mexico

**Keywords:** Biotechnology, Physiology

## Abstract

Drought-induced water stress affects the productivity of the *Vanilla planifolia* Jacks. ex Andrews crop. In vitro culture technique is an effective tool for the study of water stress tolerance mechanisms. This study aimed to evaluate the morphological, physiological and biochemical response of *V. planifolia* under in vitro water stress conditions induced with polyethylene glycol (PEG). In vitro regenerated shoots of 2 cm in length were subjected to different concentrations of PEG 6000 (0, 1, 2 and 3% w/v) using Murashige and Skoog semi-solid culture medium. At 60 days of culture, different growth variables, dry matter (DM) content, chlorophyll (Chl), soluble proteins (SP), proline (Pro), glycine betaine (GB), stomatal index (SI) and open stomata (%) were evaluated. Results showed a reduction in growth, Chl content, SP, SI and open stomata (%) with increasing PEG concentration, whereas DM, Pro and GB contents rose with increasing PEG concentration. In conclusion, PEG-induced osmotic stress allowed describing physiological and biochemical mechanisms of response to water stress. Furthermore, the determination of compatible Pro and GB osmolytes can be used as biochemical markers in future breeding programs for the early selection of water stress tolerant genotypes.

## Introduction

Vanilla (*Vanilla planifolia* Jacks. ex Andrews) is an orchid species native to Mexico used for the extraction of vanillin, one of the most economically important aromatic compounds worldwide^1^. This species, which has a hemiepiphytic habit, requires water and abundant moisture for proper development^2^. It is estimated that one of the main factors affecting vanilla production is its high susceptibility to pests and diseases and its low tolerance to water stress conditions, due to its low genetic variability^3^. In addition, various climate change scenarios forecast prolonged droughts that will cause water stress in plants^4^. Vanilla is also categorized as endangered B2ab(iii, v) version 3.1 in the International Union for Conservation of Nature and Natural Resources (IUCN) red list of threatened plants (http://www.iucnredlist.org).

In plants, the main water stress tolerance mechanisms are the accumulation of reactive oxygen species, synthesis of antioxidant enzymes, synthesis of specific proteins (hydrophilins, dehydrins, chaperonins) and osmotic adjustment mediated by compatible osmolytes^5–8^. Water stress reduces the availability of water in the cytoplasm of cells, affecting their homeostasis. Lack of water in cells causes osmotic and oxidative stress, damaging physiological, biochemical and molecular processes^5,9^.

In vitro osmotic stress simulation using osmotic agents is an efficient technique that provides a controlled and uniform environment for the study of different water stress response mechanisms^10^. High molecular weight polyethylene glycol (PEG) is highly soluble in water and non-penetrable in cells, produces a negative osmotic potential in the culture medium without generating toxicity and is one of the most widely used osmotic agents to study the effects of in vitro water stress in plants^11^. The use of PEG as an in vitro stress agent has been evaluated in crops such as sugar beet (*Beta vulgaris* L.)^12^, nopal (*Opuntia ficus-indica* (L.) Mill)^13^, wheat (*Triticum durum* Desf.)^14^, stevia (*Stevia rebaudiana* Bert.)^15,16^, and banana (*Musa acuminata* Colla)^17,18^ among others. In the family Orchidaceae, it has been used in *Acianthera teres* Lindl., *Octomeria crassifolia* Lindl., *O. gracilis* Barb. Rodr.^19^, *Prosthechea vitellina* W. E. Higgins^20^ and *Dendrobium officinale* Kimura and Migo^21^. In *Vanilla* genus there is limited information on the response to osmotic stress under in vitro conditions and the mechanisms of response to water stress in *V. planifolia* have not been fully studied. The objective of this study was to evaluate the morphological, physiological and biochemical responses in in vitro* V. planifolia* shoots under water stress conditions induced by PEG.

## Results

### PEG-induced osmotic stress

The osmotic potential (Ψs) of the culture medium rose as PEG concentrations increased, obtaining values of Ψs= − 0.14, − 0.23, − 0.32 and − 0.43 MPa for 0, 1, 2 and 3% PEG, respectively. When evaluating the effect of different PEG concentrations on the in vitro growth of *V. planifolia*, significant differences were observed in shoot length, number of leaves, number and length of roots and percentage of dry matter (Table 1).

**Table 1:** Effect of different PEG 6000 concentrations on the in vitro growth of *Vanilla planifolia*.

PEG (%)	Osmotic potential (Mpa)	Shoot length (mm)	Number of leaves	Number of roots	Root length (mm)	Fresh weight (mg)	Dry weight (mg)	Dry matter (%)
0	− 0.14	67.60 ± 3.05^a^	5.70 ± 0.30^a^	5.80 ± 0.20^a^	31.00 ± 1.59^a^	2.51 ± 0.61^a^	0.20 ± 0.04^a^	7.97 ± 0.22^b^
1	− 0.23	37.40 ± 2.0s7^b^	3.10 ± 0.23^b^	4.20 ± 0.33^b^	24.20 ± 2.55^b^	1.00 ± 0.14^ab^	0.10 ± 0.01^ab^	10.01 ± 0.61^a^
2	− 0.32	41.00 ± 3.07^b^	3.00 ± 0.26^b^	4.70 ± 0.37^b^	18.90 ± 1.21^b^	1.02 ± 0.32^ab^	0.11 ± 0.03^ab^	10.79 ± 0.69^a^
3	− 0.43	26.60 ± 0.81^c^	2.00 ± 0.21^c^	2.60 ± 0.22^c^	11.10 ± 0.64^c^	0.44 ± 0.06^b^	0.05 ± 0.00^b^	11.37 ± 0.97^a^

Values represent the mean ± SE (standard error) at 60 days of culture. Means with different letters are significantly different (Tukey, *p* ≤ 0.05).

**Figure 1:**
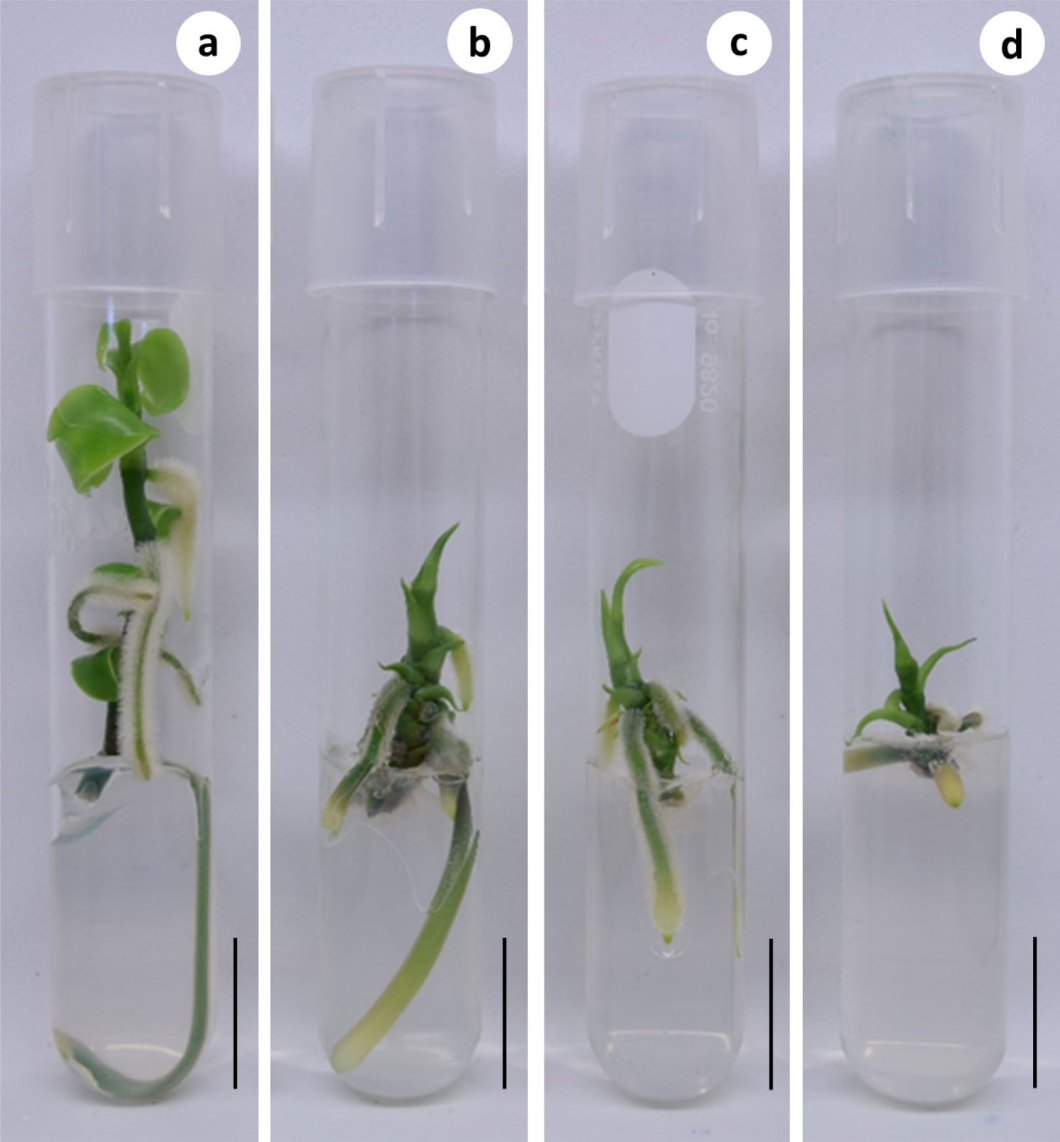
Effect of different concentrations of PEG 6000 on the growth of *Vanilla planifolia* at 60 days of culture. (**a–d**) 0, 1, 2 and 3% PEG 6000, respectively. Black bar = 2 cm.

Overall, in vitro growth of *V. planifolia* decreased with increasing PEG concentrations. The longest shoots (67.6 mm) were obtained at 0% of PEG, whereas the shortest shoots (26.6 mm) were obtained with 3% of PEG. For the variable number of leaves per shoot, the highest number of leaves (5.7) was observed in 0% of PEG, while the lowest number of leaves (2.0) was observed in 3% of PEG. For the variables number and length of roots, the shoots with the highest number (5.8) and length of roots (31.0 mm) were observed in 0% of PEG, while the smallest number (2.6) and size of roots (11.1 mm) was found in the treatment with 3% of PEG (Fig. 1). For fresh weight and dry weight, the only significant decrease (0.44 mg) and (0.05 mg) in comparison with control was recorded in 3% of PEG, respectively. Regarding dry matter content, the highest percentages (10.01, 10.79 and 11.37%) were observed in the PEG treatments, while the lowest percentage (7.97%) was observed in the control treatment.

### Chlorophyll (Chl) content

**Figure 2:**
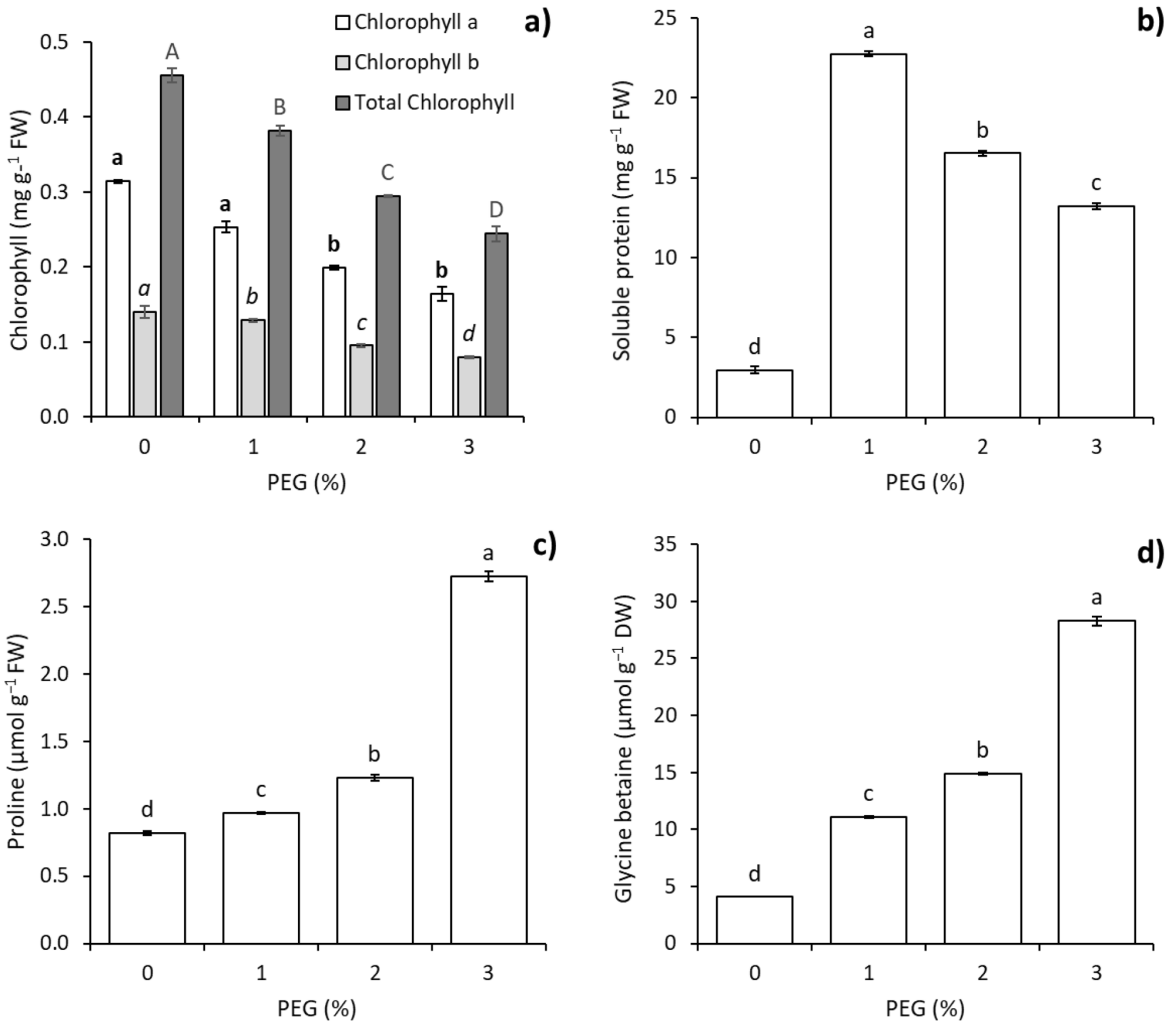
Effect of PEG 6000 on biochemical responses of in vitro* Vanilla planifolia* shoots at 60 days of culture. (**a**) Chlorophyll contents, (**b**) soluble protein, (**c**) proline and (**d**) glycine betaine. Values represent the mean ± SE (standard error). Means with different letters are significantly different (Tukey, p ≤ 0.05).

Significant differences were observed in Chl contents in leaves of *V. planifolia* seedlings exposed to different concentrations of PEG 6000. The highest total Chl (0.45 mg g^−1^ FW) and Chl a (0.32 mg g^−1^ FW) contents were observed in the 0% of PEG treatment, followed by the 1, 2 and 3% of PEG treatments, respectively. The highest Chl b contents (0.14 and 0.12 mg g^−1^ FW) were observed in the 0 and 1% PEG treatments, while lower Chl b contents (0.10 and 0.08 mg g^−1^ FW) were observed in the 2 and 3% PEG treatments (Figure 2a).

### Soluble protein (SP) content

Differences in SP content were observed at all PEG concentrations. The highest SP content (23 mg g^−1^ FW) was observed in the 1% of PEG treatment, while lower SP contents were observed (17, 14 and 3 mg g^−1^ FW) in the 2, 3 and 0% of PEG treatments, respectively (Fig. 2b).

### Proline (Pro) content

A gradual increase in Pro content was observed with increasing PEG concentration. The highest Pro content (2.7 µmol g^−1^ FW) was observed at 3% of PEG, while the lowest contents (1.2, 1.0 and 0.08 µmol g^−1^ FW) were observed in the 2, 1 and 0% of PEG treatments, respectively (Fig. 2c).

### Glycine betaine (GB) content

A gradual increase in GB content was observed with increasing PEG concentration. The highest GB content (28 µmol g^−1^ DW) was observed at 3% of PEG, while the lowest contents (15, 12 and 4 µmol g^−1^ DW) were observed in the 2, 1 and 0% of PEG treatments, respectively (Fig. 2d).

### Stomatal index (SI) and percentage of open stomata

**Figure 3:**
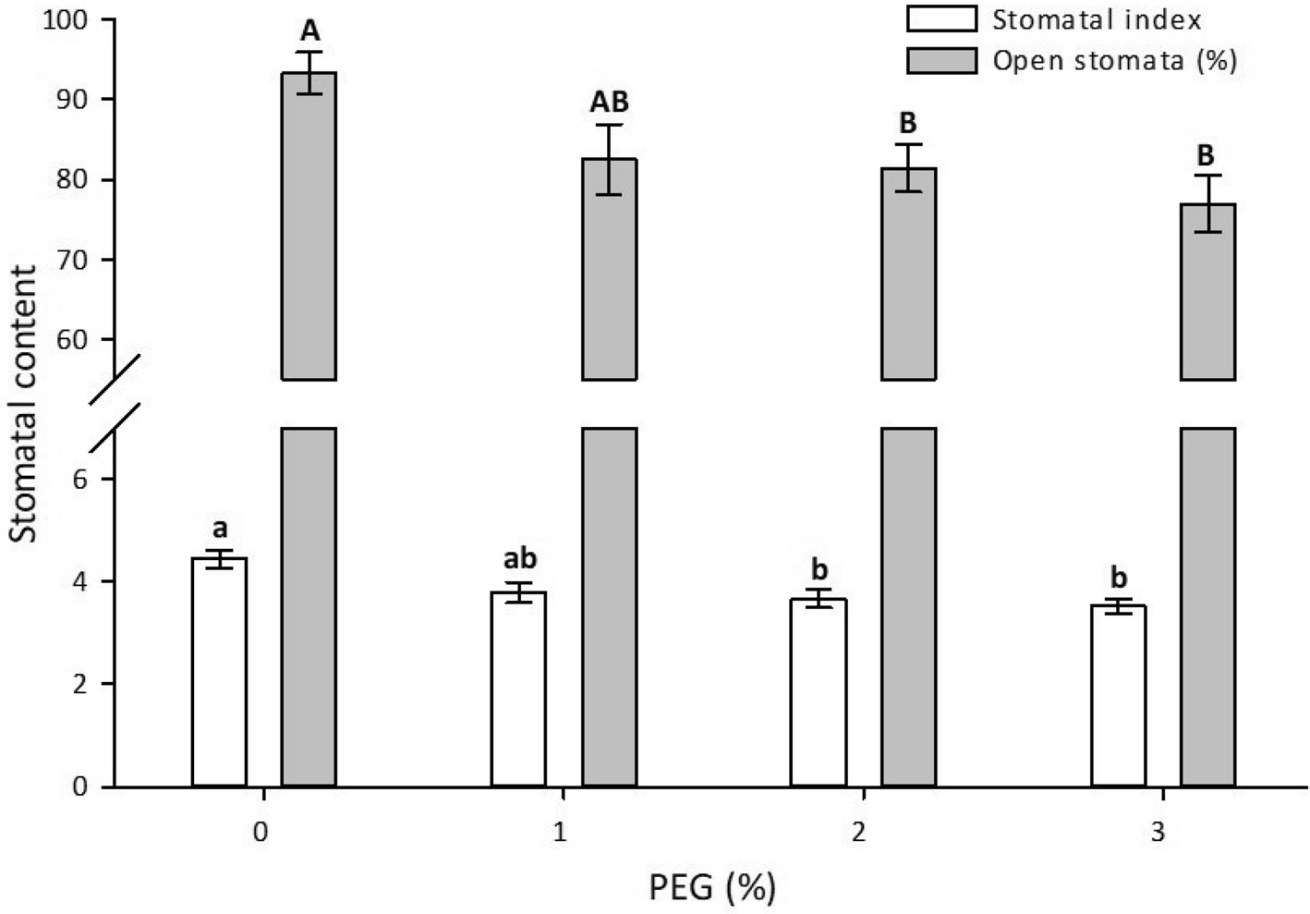
Effect of PEG 6000 on the stomatal content of in vitro* Vanilla planifolia* shoots at 60 days of culture. Values represent the mean ± SE (standard error). Means with different letters are significantly different (Tukey, p ≤ 0.05).

**Figure 4:**
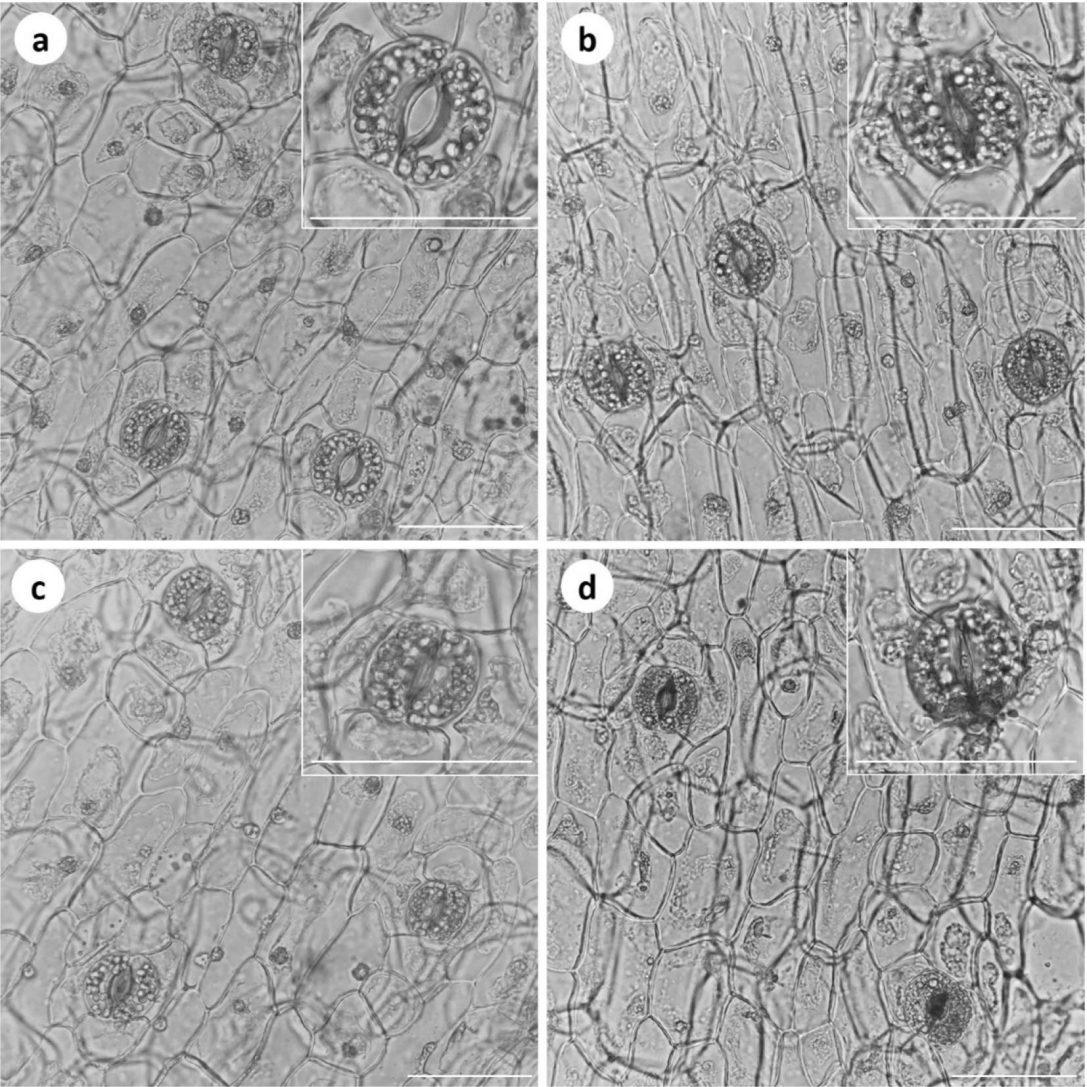
Effect of different PEG 6000 concentrations on the stomatal index and open stomata of *Vanilla planifolia*. (**a–d**) 0, 1, 2 and 3% PEG 6000, respectively. White bar = 2.5 µm.

Significant differences were observed in the SI and percentage of open stomata of *V. planifolia* exposed to different PEG concentrations. The highest SI (4.8) was observed in the control treatment, followed by the 1, 2 and 3% of PEG treatments, respectively (Fig. 3). The highest percentage of open stomata (92%) was observed in the control treatment, followed by the PEG treatments (Fig. 4).

## Discussion

Results obtained in this study demonstrate that in vitro growth of *V. planifolia* is affected under osmotic stress conditions induced by different PEG concentrations. The different PEG concentrations had an effect on the osmotic potential of the culture medium (Ψs = − 0.14, − 0.23, − 0.32 and − 0.43 MPa for 0, 1, 2 and 3% of PEG). The PEG 6000-induced osmotic stress affects the in vitro growth of *V. planifolia*. One of the most important strategies that plants have adopted against osmotic stress is to stop or slow their growth^22,23^. In fact, slow growth is an adaptive response for plant survival under stress conditions^24^.

Bello-Bello et al.^25^ reported that, during in vitro preservation of *V. planifolia* using 3% PEG 6000, this osmotic agent acts as a growth inhibitor. Similar results were described by Gao et al.^21^ who found significant differences in the growth of *Dendrobium officinale* shoots with 1, 3 and 5% of PEG 6000. Jácome-Blásquez et al.^20^ reported a reduction in germination of *Prosthechea vitellina* (Lindl.) W. E. Higgins with an increasing concentration of PEG 8000. Ramírez-Mosqueda et al.^26^ obtained the lowest number of leaves and roots per explant in *Laelia anceps* Lindl. with 3% of PEG 8000. Piwowarczyk et al.^27^ reported an increased dry matter content in shoots of eight genotypes of grass pea (*Lathyrus sativus*) with 5, 10 and 15% of PEG 6000. According to Rai et al.^28^, growth inhibition under water stress is associated with a reduction in the ability of plants to absorb water and nutrients caused by the decreased water potential of the culture medium.

In this study, the inhibition of in vitro* V. planifolia* shoot growth in the presence of PEG could be explained by the synthesis of phytohormones responsible of stomatal closure, causing a reduction in transpiration and low carbon assimilation capacity. According to the authors of Ref.^29^, the stomatal closure under drought condition is due to the accumulation of compounds such as abscisic acid, methyl jasmonate, ethylene, and brassinosteroids. On the other hand, the increase in dry matter content in the presence of PEG could be explained by synthesis of specific proteins, compatible osmolytes and/or metabolites in response to water stress and probably due to accumulation of inorganic solutes in relation to osmotic adjustment.

In this study, a decrease in total Chl content was observed in *V. planifolia* shoots in relation to the increase in PEG concentration. In Orchidaceae in vitro, Gao et al.^21^ reported a decrease in Chl content in *D. officinale* shoots exposed to 0, 1, 3 and 5% of PEG 6000. Suis et al.^30^ found a reduction in Chl content in protocorm-like bodies of *Aranda* Broga Blue Bell with 15% of PEG 400. A reduction in Chl content in *V. planifolia* under water stress (− 0.9 MPa) has been reported under ex vitro conditions^31^. For chlorophyll content, according to the authors of Ref.^32^, a reduced chlorophyll content under water stress is caused by a decrease in photosynthetic activity due to stomatal closure. Also, the low photosynthetic activity is related to dry matter accumulation as a result of osmotic stress affecting carbohydrate transport^33,34^. In addition, with low photosynthetic activity, Chl degradation is a response mechanism to avoid energy wasting^35–37^.

Results of biochemical analysis showed significant differences in SP, Pro and GB contents (Fig. 2). The SP content decreased with increasing PEG concentration in *V. planifolia* shoots; however, SP content was significantly higher in all PEG treatments with respect to the control treatment. Variation in SP content is a water stress tolerance response^37^ related to synthesis, accumulation or degradation. The SP accumulation at 1% of PEG compared to control (without PEG stress) is probably related to the synthesis of hydrophilins^38^, aquaporins^39^, dehydrins^40^, antioxidant enzymes^41^, and chaperonins^42^, among others. Plants can synthesize this type of protein to neutralize oxidative stress and osmotic stress^41,43^. The SP reduction in 2 and 3% of PEG could be explained due to a degradation and/or synthesis reduction caused by a physiological wilting point in plants during in vitro osmotic stress.

Qayyum et al.^44^ reported an increase in protein content in seedlings of five wheat (*Tritricum aestivum* L.) cultivars exposed to different osmotic potentials (− 0.2, − 0.4 and − 0.6 MPa) generated with PEG 6000. In contrast, Razavizadeh et al.^35^ observed a decrease in protein content in thyme (*Thymus vulgaris*) seedlings grown under 2, 4, 6 and 8% of PEG 6000. Similarly, Gao et al.^21^ observed a decrease in protein content in *D. officinale* shoots grown under water stress induced with 1, 3 and 5% of PEG 6000. Suis et al.^30^ observed a reduction in SP using PEG 400 (0, 5, 10 and 15%) in protocorm-like bodies of the *Aranda* Broga Blue Bell orchid. Chandran and Puthur^31^ note that the decrease in SP content in the V4 somaclone of *V. planifolia* under water stress (− 0.9 MPa) may be due to its degradation.

The results of this study suggest that under the in vitro conditions evaluated, protein accumulation in 1% of PEG treatments could be explained by the biosynthesis of specific proteins as a mechanism of tolerance to osmotic stress; whereas, the decrease in SP content at doses of 2 and 3% of PEG could be due to a reduction in biosynthesis and/or an increase in their degradation. Razavizadeh et al.^35^ note that protein degradation under osmotic stress provides sources of nitrogen used in different metabolic processes.

The Pro accumulation in plants is a physiological response to salt and water stress by reducing plant growth^45^. Under water stress conditions, Pro acts as a stabilizer of structural proteins, a compatible osmolyte and a catalyst of antioxidant enzyme activity^46^. In our study, a significant increase in Pro content was observed in shoots exposed to PEG; however, the 3% PEG treatment showed a twofold increase in Pro content compared to the control. This result suggest that Pro has a role in water stress tolerance mechanisms in *V. planifolia*.

Other studies show that Pro is a biochemical indicator that contributes to water stress tolerance mechanisms. Piwowarczyk et al.^27^ reported an increase in Pro content in grass pea (*Lathyrus sativus*) shoots at 5, 10 and 15% of PEG 6000. Razavizadeh et al.^35^ observed an increase in Pro accumulation in thyme (*Thymus vulgaris*) shoots at 2, 4, 6 and 8% of PEG. In Orchidaceae, Jácome-Blásquez et al.^20^ found that the Pro content in *Prosthechea vitellina* (Lindl.) W. E. Higgins increased by twice as much relative to the control treatment by increasing the PEG 8000 concentration. In contrast, a previous study reported^21^ that the Pro content in *Dendrobium officinale* shoots decreased in the treatments with 1, 3 and 5% of PEG 6000. In addition to Pro, there are other osmolytes such as sugars and GB that accumulate in response to water stress^47^.

Glycine betaine content showed a significant increase when *V. planifolia* shoots were exposed to PEG. To date, GB accumulation in *V. planifolia* under in vitro water stress has not been reported. However, GB accumulation has been reported in other species. Hajihashemi and Ehsanpour^48^ reported GB accumulation in *S. rebaudiana* shoots exposed to 2, 4 and 6% of PEG 6000. Datir and Inamdar^49^ observed that GB accumulation increased in all wheat (*T. aestivum* L.) cultivars in response to water stress induced by 15% of PEG 6000. Jácome-Blásquez et al.^20^ found increased GB content in *P. vitellina* (Lindl.) W. E. Higgins at 5–15% of PEG 8000. In addition, GB is a compatible osmolyte that acts as an osmoprotectant, maintains photosynthetic activity by protecting the thylakoid membrane, and neutralizes reactive oxygen species by regulating the expression of oxidative stress response genes during osmotic stress^37,50–52^. The absence of GB in cells could result in cell membrane damage, protein degradation, DNA damage and loss of photosynthetic capacity^37,53,54^.

PEG-induced osmotic stress reduced the SI and percentage of open stomata in *V. planifolia*. Paletri et al.^55^ reported a decrease in SI in *Cattleya* sp. Lindl. in vitro with 25% of PEG 6000. Chandran and Puthur^31^ reported a reduced SI (1.65) in ex vitro somaclones of *V. planifolia* under water stress generated by water deficit (− 0.9 MPa). According to the authors of Ref.^56^, variation in the SI under water stress is different for each species. *V. planifolia* under normal conditions has a SI of 2.81 ± 0.27^31^. On the other hand, stomatal closure as a result of using PEG for the evaluation of osmotic stress in vitro has been reported. Another study^57^ found a reduction in stomatal opening in Persian walnut (*Juglans regia* L.) of 43 and 58% at 3 and 5% of PEG 6000 relative to the control.

Plant stomata are normally open during in vitro conditions due to excess water in culture medium and high humidity within the culture vessels. However, the stomatal functionality is stimulated during in vitro PEG-induced osmotic stress. Stomatal functionality, through the opening and closing of stomata, is a mechanism that regulates transpiration and maintains water balance during hydric stress^58^. In this regard, it has been reported^59^ that adequate stomatal functionality regulates the water balance in plants. Stomatal closure and a low SI generate a low transpiration rate to avoid dehydration due to water loss.

## Methods

### Plant material selection and in vitro establishment

*Vanilla planifolia* Jacks. ex Andrews has been listed in the IUCN due to its highly fragmented populations. For sampling and in vitro propagation of plant material, an appropriate permission was granted by the National Agro-Alimentary Health, Safety and Quality Service (SENASICA, certificate No. LAB 30044001/2018) and the authorization approved by land or facilities that manage wildlife (No. SGPA/DGVS/2868/19). All procedures performed in the study were in compliance with the IUCN Policy Statement on Research Involving Species at Risk of Extinction and the Convention on the Trade in Endangered Species of Wild Fauna and Flora. For in vitro establishment, young *V. planifolia* cuttings with 7–8 buds were collected from a commercial plantation located in the community of El Palmar, Emiliano Zapata, Veracruz, Mexico (19° 39′ 86′′ N and − 96° 75′ 17′′ W). Cuttings were kept in quarantine under greenhouse conditions at an irradiance of 130 μmol m^−2^ s^−1^, 30 ± 2 °C, and 60 ± 5% relative humidity, in potting soil (peatmoss and agrolite, 1:1) with weekly applications of 0.1% (w/v) fungicidal solution (Cupravit, Bayer AG, Leverkusen, NW, DEU) and 0.1% (w/v) bactericidal solution (Agrimycin, Pfizer, New York, NY, USA). After 40 days, 3–5 cm long nodal segments were collected. The nodes were transferred to the laboratory where they were washed with Axion commercial detergent (Mission Hills, S.A. de C.V., San José de Iturbide, GT, MEX) and tap water; subsequently, they were reduced to 2 cm in length and immersed in a soap solution with two drops of Tween 20 (Sigma-Aldrich Chemical Company, St. Louis, MO, USA) per 100 mL for 5 min followed by five rinses with distilled water. In a laminar flow hood, the explants were immersed in a 15% (v/v) solution of commercial NaClO (5% AI, Clorox, Monterrey, NL, MEX) solution and three rinses were performed with sterile distilled water. Subsequently, the explants were immersed in a 0.1% (w/v) of mercuric chloride (HgCl_2_) solution and three rinses were performed with sterile distilled water. Finally, the disinfected explants were individually transferred to 500 mL flasks containing 30 mL of MS^60^ multiplication medium supplemented with 30 g L^−1^ sucrose, 2 mg L^−1^ 6-Benzylaminopurine (BAP) (Sigma-Aldrich) and 2.3 g L^−1^ Phytagel (Sigma-Aldrich) as a gelling agent. The pH of the medium was adjusted to 5.8 and autoclaved for 15 min at 121 °C and 117.7 kPa. All cultures were incubated at 24 ± 2 °C with a 16 h photoperiod with fluorescent lamps at an irradiance of 50 μmol m^−2^ s^−1^.

### Osmotic stress induction with PEG

For in vitro selection pressure with PEG, 2 cm long shoots (without roots) obtained after four subcultures of 45 days each in multiplication medium were used. The shoots were grown in 22 × 150 mm test tubes containing 20 mL of MS semi-solid medium without growth regulators and different concentrations of PEG 6000 MW (Sigma-Aldrich) (0, 1, 2 and 3% w/v). The PEG was added directly into the culture medium. The culture medium pH and the sterilization and incubation conditions were the same as described above. Each treatment consisted of 10 explants, with one shoot per test tube. At 60 days of culture (without explant subcultures), the different morphological variables and percentage of dry matter (DM) were evaluated. The DM content was calculated using the formula: dry weight/fresh weight × 100. Chl, SP, Pro and GB contents were determined. Additionally, SI and percentage of open stomata were evaluated.

### Osmotic potential measurements of the culture medium

The osmotic potential (Ψs) of the semi-solid culture media with different PEG concentrations was determined using a vapor pressure osmometer (5520 Vapro, Wescor Inc., Logan, UT, USA) at the beginning of osmotic stress induction.

### Determination of chlorophyll (Chl) content

The Chl content was determined according to the methodology proposed by Ref.^61^ and with slight modifications by Ref.^62^. The absorbance readings were measured in a spectrophotometer (Genesys 10S, Thermo Scientific, Waltham, MA, USA) at 663 nm and 645 for Chl a and b, respectively. The Chl content was calculated using the following formulas:

$$ {\text{Chlorophyll a }}\left( {\text{C}} \right) \, = \, \left( {[({12}.{7 } \times {\text{A}}_{{{663}}} ) - ({2}.{59 } \times {\text{A}}_{{{645}}} )] \, \left( {\text{V}} \right)} \right)/({1}000 \, \times {\text{W}}), $$

$$ {\text{Chlorophyll b }}\left( {\text{C}} \right) \, = \, \left( {[({22}.{9 } \times {\text{A}}_{{{645}}} ) - ({4}.{7}0 \, \times {\text{A}}_{{{663}}} )] \, \left( {\text{V}} \right)} \right)/({1}000 \, \times {\text{W}}), $$

$$ {\text{Total Chl }}\left( {\text{C}} \right) \, = \, \left( {[({8}.{2}0 \, \times {\text{A}}_{{{663}}} ) \, - \, ({2}0.{2 } \times {\text{A}}_{{{645}}} )] \, \left( {\text{V}} \right)} \right)/\left( {{1}000 \, \times {\text{ W}}} \right). $$

where A_663_ and A_645_: Absorbance; C: Concentration (mg g^−1^ FW); V: volume graduation in mL^−1^; W: sample weight in g; 1000: conversion factor.

### Determination of soluble protein (SP) content

Soluble protein estimation was carried out with the colorimetric method proposed by Bradford^63^. First, 100 mg of fresh leaf tissue were weighed and macerated in a mortar in 25 mL of cold acetone for 5 s. The macerated tissue was vacuum filtered and 12.5 mL of acetone was added. Then 5 mL of 0.1M TRIS–HCl Buffer (pH 7.1) was added to the extract obtained and the samples were stored on ice bath. Subsequently, they were centrifuged at 3100×*g* for 20 min at 4 °C. A 100 µL aliquot of the supernatant was acquired and added to 4.9 mL of Bradford reagent. After 12 min of reaction, the chromophore was read at an absorbance of 595 nm in a spectrophotometer (Genesys 10S). Quantification was done using a calibration curve with bovine albumin standard (Hoffmann-La Roche Ltd., Grenzacherstrasse, BS, SWI).

### Determination of proline (Pro) content

Proline determination was carried out by the method described by Ref.^64^. First, 125 mg of fresh leaf tissue were macerated in a mortar and homogenized with 5 mL of 3% (w/v) sulfosalicylic acid. The resulting solution was filtered through Whatman #2 filter paper. Then 1 mL aliquot was acquired, 1 mL of glacial acetic acid and 1 mL of ninhydrin were added. Incubation of 1 mL of the extract was carried out in a thermoregulated bath for one hour at 100 °C. The tubes were removed and the reaction terminated in an ice bath. Then 2 mL of toluene was added and mixed for 30 s for phase separation. The resulting chromophore was read at an absorbance of 520 nm in the spectrophotometer (Genesys 10S) using toluene as a blank. Quantification was done using a calibration curve with l-proline standard (Sigma-Aldrich).

### Determination of glycine betaine (GB) content

Glycine betaine determination was carried out by the method proposed by Ref.^65^. For this, 100 mg of dry leaf tissue were weighed and reacted with water and 2N H_2_SO_4_ after the addition of 0.2 mL of periodide complex (KI-I_2_). Extraction was performed by centrifugation at 3100×*g* for 20 min at 4 °C and subsequent addition of 9 mL of 1,2-Dichloroethane. Absorbance was measured at 365 nm in a spectrophotometer (Genesys 10S) using 1,2-Dichloroethane as a blank. Quantification was done using a calibration curve with betaine standard (Sigma-Aldrich).

### Stomatal index (SI) and percentage of open stomata

The SI and the percentage of open stomata were measured on the underside of the third leaf in relation to the shoot apex. The SI was determined by the formula: SI = [number of stomata/(number of epidermal cells + number of stomata)] × 100. To visualize the stomata and epidermal cells per mm^2^, leaf samples were examined under a microscope (Axio Lab.A1, Carl Zeiss AG, Jena, TH, DEU). The percentage of open stomata was calculated using the total number of stomata and the number of open stomata.

### Experimental design and statistical analysis

All experiments were conducted with a completely randomized design and replicated three times (3 × 10), with a total of 30 explants in each treatment. The data obtained were tested with an analysis of variance (ANOVA) followed by Tukey’s test (*p* ≤ 0.05), performed using IBM SPSS statistical software version 22 (https://www.ibm.com/support/pages/node/313621).

## Conclusions

Polyethylene glycol induced osmotic stress under in vitro conditions had effects on growth, physiology and biochemical determinations evaluated in *V. planifolia*. In this study, a reduction in growth, chlorophyll content, soluble proteins, stomatal index and open stomata (%) was observed with increasing PEG concentration, whereas dry matter, proline and glycine betaine contents rose with increasing PEG concentration. The compatible proline and glycine betaine osmolytes will be helpful mechanism of water stress tolerance in *V. planifolia* genotypes. In addition, ROS production and antioxidant enzyme activities could be evaluated in a future study for a better understanding of water stress tolerance. Further research is recommended to study others species in *Vanilla* genus in these in vitro conditions.
